# Datasets of narrow thermal hysteresis behaviour Ti-Ni-based HT-SMAs and the predicted accumulated local effects

**DOI:** 10.1016/j.dib.2023.109654

**Published:** 2023-10-13

**Authors:** Ronald Machaka, Precious M. Radingoana

**Affiliations:** a Independent Researcher, Idya Factory Co., Hamilton, New South Wales 2303, Australia; b Independent Researcher, Pretoria, 0001, South Africa

**Keywords:** High-temperature shape memory alloys, HT-SMA, Ti-Ni-based HT-SMA, Narrow thermal hysteresis, Explainable machine learning, Accumulated local effect

## Abstract

This article refers to data derived from a research article entitled “Prediction of narrow HT-SMA thermal hysteresis behaviour using explainable machine learning” [1]. It is based on the knowledge that alloying Ti-Ni-based shape memory alloys (SMAs) with additional ternary or multicomponent elements can alter the SMAs’ characteristic transformation temperatures, including the thermal hystereses. Two datasets are reported. The first and primary dataset documents experimental Ti-Ni-based shape memory alloys’ high-transformation temperature characteristics reported in the literature. The second auxiliary dataset presented in this article was obtained following the explainable prediction of the narrow high-temperature thermal hysteresis behaviour in Ti-Ni-based high-transformation temperature SMAs (HT-SMAs). The second dataset is intended to generalise and summarise the ML prediction and visualisation of the thermal hysteresis behaviour as also observed experimentally in multiple reports elsewhere.

The datasets are provided as supplementary files and the second dataset is also visualised as an intuitive marginal effects plot. We believe that these data will find applications in advancing experimental and theoretical HT-SMA research.

Specifications Table

**Table:** 

Subject	Materials Science
Specific subject area	High-temperature shape memory alloys
Type of data	The datasets reported in this article have the following types: *i. Tables &* Fig*ures*
How the data were acquired	The main dataset was collected from experimental observations reported in peer-reviewed research articles. The auxiliary dataset reported in this article was acquired following the prediction of narrow thermal hysteresis behaviour in Ti-Ni-based HT-SMAs using explainable machine learning and subsequent model-agnostic methodologies, namely accumulated local effects (ALE), and the SHapley Additive exPlanations (SHAP). A custom-built deep learning GPU workstation was used to acquire the auxiliary dataset. Its basic technical specifications are as follows: i. Hardware: *(a) CPU: AMD Ryzen 7 5800×8-core, 16-Thread Elite Gaming* *(b) GPU: 1 X Nvidia GeForce RXT 3060 Gaming X 12G (12GB of GDDR6X VRAM, 3584 CUDA cores, and 28 tensor cores)* *(c) RAM: 4×12GB DDR4 sticks* *(d) PSU: 1200-watt power supply unit* ii. Workstation OS: *(a) Long-Term Support Ubuntu 22.04 (Jammy Jellyfish)* iii. Python virtual environment: *(a) [Python 3.9, Nvidia driver v515, CUDA Toolkit v11.7 toolkit and cuDNN 8.5 installed]* (b) *[PyALE [2] and other compatible ML packages installed]*
Data format	The data reported in this article are in the following formats: *i. Raw, tabulated and plotted*
Description of data collection	Two datasets are presented; the main dataset comprises secondary data and the auxiliary dataset is simulated. The main dataset was collected from experimental observations reported in peer-reviewed research articles: The following data entries were captured: *i. Identification of experimental Ti-Ni-based alloy, e.g. TiNi,* *ii. Specification of the Ti-Ni-based alloys’ multicomponent elemental composition (in at. %)* *iii. The reported characteristic transformation temperatures,* *iv. The derived narrow thermal hysteresis characteristics* The additional data reported are not secondary data. The data were acquired following a machine learning computation using the above-described hardware and software. In summary: *(a)* *An experimental data-informed ML-based XG-Boost model was developed using the Scikit-Learn and XGBoost libraries in the Python 3.9 environment. The model predicts and explains the narrow hysteresis behaviour in Ti-Ni HT-SMAs.*The model only takes the alloys’ multicomponent elemental composition as inputs (x-axis). *(b)* *Following the model development, the ALE analysis technique was implemented, also in the Python 3.9 environment, to analyze the marginal effects of each element's composition on the narrow hysteresis behaviour.The implementation of the ALE method produces a dataset containing the expected change in ML predicted ΔT values and the marginal effects of the nine selected factors (i.e. Ti, Ni, Pd, Pt, V, Hf, Zr, Cu, and Co)* *(c)* *The dataset has nine sets of simulated samples. More specifically, The y-axis is the predicated expected change in ML predicted ΔT value that affects the predictions of the model (i.e., predicted ΔT) while holding the values of all the other factors constant.*
	ii. Lastly, ALE plots were generated and validated/interpreted according to prior experimental findings, e.g. [3 - 14].
Data source location	The data reported are stored on the cloud (ISP withheld) and physically at: *i. Institution: Idya Factory Co. (Independent Research)* *ii. City/Town/Region: Newcastle, New South Wales 2303* iii. *Country: Australia*
Data accessibility	All data referred to in your data article are publicly available: • Repository name: Mendeley Data • Data identification number: **DOI:** 10.17632/cj4hc9f2g4.3
Related research article	Ronald Machaka, Precious M. Radingoana, Prediction of narrow HT-SMA thermal hysteresis behaviour using explainable machine learning, Mater. Today Commun. 35 (2023) 105806. https://doi.org/10.1016/j.mtcomm.2023.105806

## 1 Value of the Data

The value of the data is summarised below:

• The main dataset is valuable because it consolidates significant prior published data on narrow HT-SMA thermal hysteresis behaviour;
• The second dataset is valuable because it improves the generalized understanding of the influence of the Ti-, Ni-, Pd-, Pt-, V-, Hf-, Zr-, Cu-, and Co- contents have on the narrow HT-SMA thermal hysteresis behaviour
• Some materials used in the development of Ti-Ni-based HT-SMAs are rare-precious metals that are scarce and prohibitively expensive, researchers working on HT-SMA development can significantly benefit from the availability of these consolidated and simulated datasets.
• As demonstrated in the original paper [[1], [2]], these simulated data agree with prior published data, the datasets will be used to investigate and validate newly proposed narrow HT-SMA candidates.
• Finally, these datasets are part of a case study curated to demonstrate the benefits of interpreting ML-predicted outcomes in the advanced materials and engineering field.

## 2 Objective

The work grows from a need to develop prediction models that are not only accurate but explainable from a practical metallurgical persuasion. The objective of the work was to produce results and outcomes that communicate usable knowledge to the advanced materials and metallurgical community. The data reported in this article are intended for practitioners – those researchers busy planning, conducting, or interpreting their own experimental or computational work. The consolidated main dataset will find applications in advancing experimental and theoretical HT-SMA developments while the simulated dataset will summarise our findings [1] to those readers – without having them leave their work to try and understand ML, posthoc models, or underlying ‘codework’.

## 3 Data Description

**Fig. 1:**
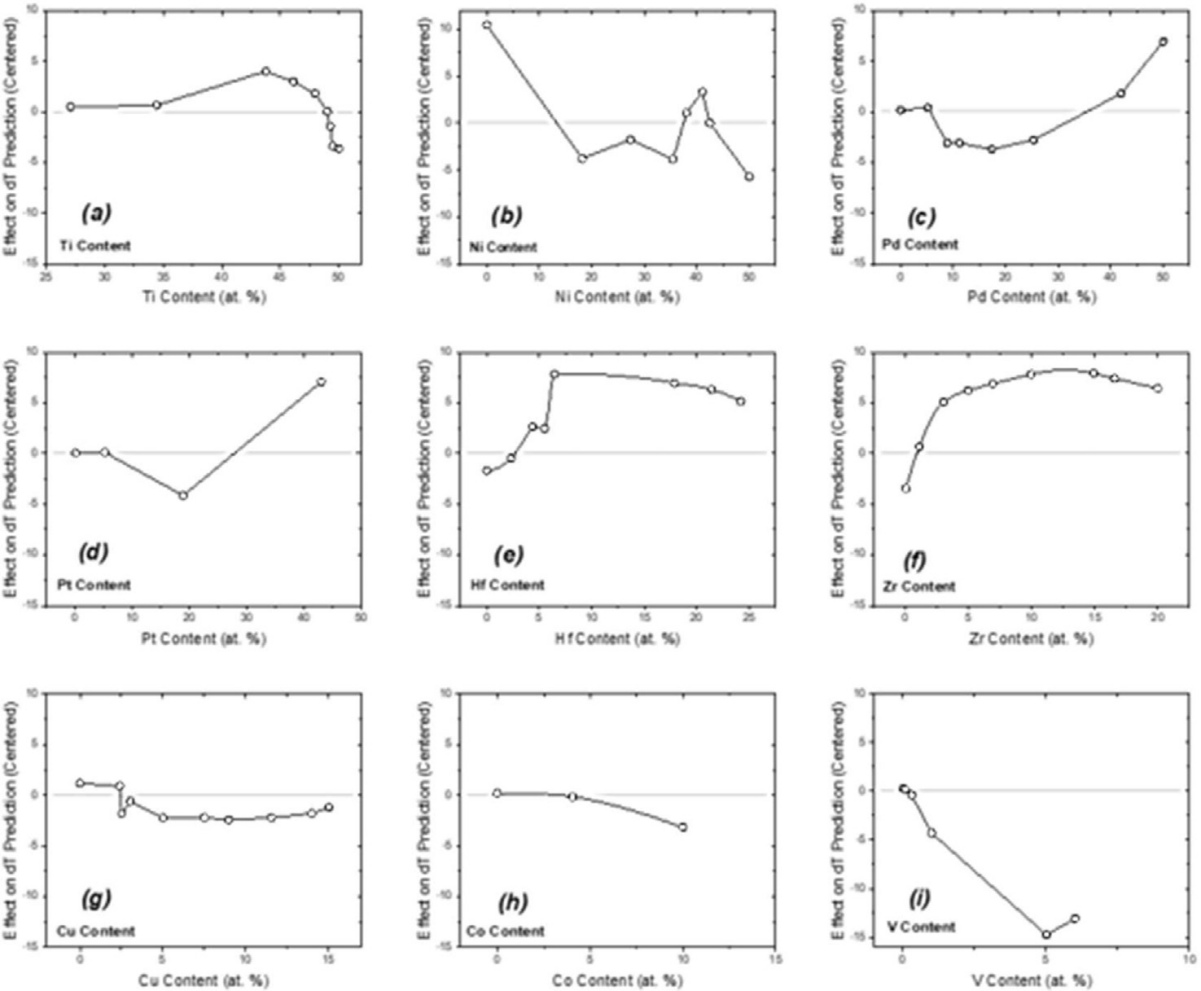
The first-order ALE main effects plots showing the influences of the alloys’ elemental on the HT-SMA hysteresis predicted behaviour [4].

The data presented in this article are divided into two components. That is, (i) the main dataset which consolidates significant prior published data on narrow HT-SMA thermal hysteresis behaviour of novel Ti-Ni-based HT-SMAs, (ii) an auxiliary dataset simulated based on the related research article findings [1,3], and a figure (Fig. 1) summarising the auxiliary dataset. The data are included as data files. The description of these files has been provided in Table 1.

**Table 1:** The list of all data files included.

Filename	Description
Processed_RawData_v2d. CSV	The main dataset consolidates significant prior published data on narrow HT-SMA thermal hysteresis behaviour of novel Ti-Ni-based HT-SMAs. The dataset reported here has seventeen columns and 190 entries. *i. Column 1 corresponds to the identification of the dataset entry (HTSMA_exp_0000).* *ii. Columns 2 to 12 capture composition-specific features (in at. %), i.e. the specification of the alloys’ elemental compositions* *iii. Columns 13 to 16 correspond to MT temperatures (Ms, Mf, As, Af). Column 17 captures the measured thermal hysteresis behaviour* iv. *All other features such as the alloy processing and post-processing parameters and multi-component alloy empirical design parameters (such as the enthalpy of mixing – dHmix, valence electron concentration – VEC, and atomic size difference, δ) are intentionally removed.*
dT_HTSMA_ML_Data_v3c.DOCX	The second dataset is simulated based on an ML model. The dataset has nine tables and figures: *i. Each table captures the data on the effect of each alloying element -on the predicted thermal hysteresis behaviour, ΔT* ii. *Each ALE plot visualizes data captured in the corresponding table*
ALE plots	The dataset is also visualized here in the form of a main-effects type first-order ALE plot (see Fig. 1). The ALE plot has nine subplots; each subplot is an x-y data sample wherein the x variables are the changing content of an alloying element (in at. %) and the y variable is the effect that change has on the predicted ΔT value. Therefore, each subplot captures the narrowing (downward) or widening (upward) trends each alloying element has on the predicted thermal hysteresis behaviour [5].

## 4 Experimental Design and Methods

The main dataset is constructed from HT-SMA thermal hysteresis behaviour observations reported in experimental peer-reviewed research reports. It also builds upon data published by Yamabe-Mitarai [6], Frenzel, et al. [7], and Ma, Karaman, and Noebe [8]. The original dataset has at least 297 alloys. After removing non Ti-Ni-based HT-SMAs, entries missing some data, and eliminating alloys with wider HT-SMA hysteresis behaviour (> 60 °C), the constructed dataset is reduced to 190 Ti-Ni HT-SMAs.

The reported auxiliary data are simulated, and not necessarily secondary data; the data were acquired following machine learning and post-hoc ALE computations using the above-described hardware and software.

**Fig. 2:**
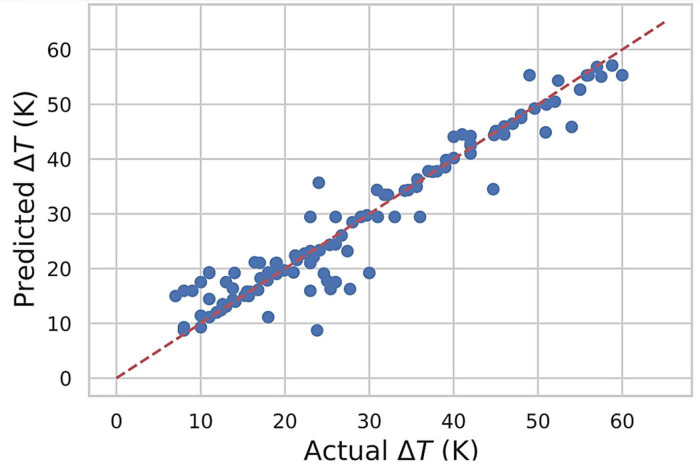
Regression scatter plot for the predicting ML model; The x-axis represents the actual ΔT values and the y-axis the predicted ΔT values. The dashed red line has diagonal parity and a slope of 1.
*(d) The ALE post-hoc analysis technique was implemented in the Python environment using the PyALE package to analyze the marginal effects of each element's composition on the narrow hysteresis behaviour. The implementation of the ALE method produces a dataset showing how see how the ML prediction (y-axis) changes as a function of the predictor features (x-axis) i.e., Ti-, Ni-, Pd-, Pt-, V-, Hf-, Zr-, Cu-, and Co- amounts in the alloy. see*Fig. 1*.*
*(e) The dataset has nine sets of simulated samples. More specifically, the y-axis is the predicated expected change in ML predicted ΔT value that affects the predictions of the model (i.e., predicted ΔT) while holding the values of all the other compositions constant.*

*(a) An ML-based XG-Boost model was developed using the Scikit-Learn and XGBoost libraries in the Python environment.*
*(b) The model inputs data collected from experimental reports in the public domain to predict the narrow hysteresis behaviour (< 60 °C) in novel Ti-Ni HT-SMAs. The data is structured as a dataframe herein each row defines an alloy's multicomponent elemental composition as taught in our previous work [*3*].*
*(c) The ML model development and implementation involves the prediction of the narrow hysteresis behaviour in novel Ti-Ni HT-SMA. The model follows a conventional ML-supervised regression where the accuracy of the model is assessed by comparing the ML predictions against true (previously known) values. See*Fig. 2*.*

Lastly, ALE plots (see Fig. 1) were generated and validated/interpreted according to prior experimental findings, e.g. [[4], [6], [7], [8], [9], [10], [11], [12], [13], [14], [15], [16], [17]].

## Ethics Statement

The research work did not involve human or animal subjects or the collection of data from social media or similar platforms. The use of artificial intelligence (AI) tools reported in this article, and the original study, is responsible and in line with the highest ethical standards including Australia's AI Ethics Principles [18]. None of the data were collected from any social media platforms.

## CRediT Author Statement

R. Machaka (RM): Conceptualization (lead), Funding acquisition (lead), Data curation (supporting), Methodology (lead), Hardware & computations (lead), Visualisation (lead), Writing – original draft (lead), Writing – review & editing (equal). P.M. Radingoana (PMR): Data collection (lead), Methodology (supporting), formal analysis (equal), Writing – review & editing (equal).

## Data Availability

Prediction of narrow HT-SMA thermal hysteresis using machine learning: A data file (Original data) (Mendeley Data).

## Appendix Supplementary materials

Image, application 1

## References

[1] Machaka R., Radingoana Precious M (2023). Prediction of narrow HT-SMA thermal hysteresis behaviour using explainable machine learning. Mater. Today Commun..

[2] Ronald Machaka, Precious M. Radingoana, ‘Corrigendum to “Prediction of narrow HT-SMA thermal hysteresis behaviour using explainable machine learning” [Mater. Today Commun. 35 (2023) 105806]’, *Mater. Today Commun.*, In Press, p. 107112, doi: 10.1016/j.mtcomm.2023.107112.

[3] Machaka R., Motsi G.T., Raganya L.M., Radingoana P.M., Chikosha S. (2021). Machine learning-based prediction of phases in high-entropy alloys: a data article. Data Brief.

[4] Zhang Z., James R.D., Müller S. (2009). Energy barriers and hysteresis in martensitic phase transformations. Acta Mater..

[5] Machaka Ronald (2023). “Prediction of narrow HT-SMA thermal hysteresis using machine learning: a data file”. Mendeley Data.

[6] Yamabe-Mitarai Y. (2020). TiPd- and TiPt-based high-temperature shape memory alloys: a review on recent advances. Metals.

[7] Frenzel J., Wieczorek A., Opahle I., Maaß B., Drautz R., Eggeler G. (2015). On the effect of alloy composition on martensite start temperatures and latent heats in Ni–Ti-based shape memory alloys. Acta Mater..

[8] Ma J., Karaman I., Noebe R.D (2010). High temperature, shape memory alloys. Int. Mater. Rev..

[9] Apley Daniel W., Zhu Jingyu (2020). Visualizing the effects of predictor variables in black box supervised learning models. J. R. Stat. Soc. Ser. B, R. Stat. Soc..

[10] Karaca H.E., Saghaian S.M., Ded G., Tobe H., Basaran B., Maier H.J., Noebe R.D., Chumlyakov Y.I. (2013). Effects of nanoprecipitation on the shape memory and material properties of an Ni-rich NiTiHf high temperature shape memory alloy. Acta Mater..

[11] Ramaiah K.V., Saikrishna C.N., Gouthama S.K.Bhaumik (2014). Ni24.7Ti50.3Pd25.0 high temperature shape memory alloy with narrow thermal hysteresis and high thermal stability. Mater. Des..

[12] Klopotov A., Gunther V., Marchenko E., Baigonakova G., Chekalkin T., Kim J., Kang J. (2017). Impact of annealing temperature on martensite transformations and structure of quaternary Ti50Ni47.7Mo0.3V2 alloy. Adv. Mater. Lett..

[13] Bigelow G.S., Benafan O., Garg A., Noebe R.D. (2021). Effect of Hf/Zr ratio on shape memory properties of high temperature Ni50.3Ti29.7(Hf,Zr)20 alloys. Scr. Mater..

[14] Yang X., Ma L., Shang J. (2019). Martensitic transformation of Ti50(Ni50−xCux) and Ni50(Ti50−xZrx) shape-memory alloys. Sci. Rep..

[15] Miyazaki S., Ishida A. (1999). Martensitic transformation and shape memory behavior in sputter-deposited TiNi-base thin films. Mater. Sci. Eng. A.

[16] Mohammed S.H., Aljubouri A.A., Mohammed M.A. (2020). The effect of cobalt element addition on the characteristics of equiatomic NiTi shape memory alloy. J. Phys. Conf. Ser..

[17] Mao H., Yang H., Shi X., Li Y., Zhang J., Jiang J. (2018). Transformation and superelastic characteristics of large hysteresis TiNi matrix shape memory alloys reinforced by V nanowires. Mater. Lett..

[18] DISR, Department of Industry, Science and Resources, Australia's AI Ethics Principles (2019). Department of industry, science and resources. Retrieved April 8, 2023, from https://www.industry.gov.au/publications/australias-artificial-intelligence-ethics-framework/australias-ai-ethics-principles.

