# Supplementary material for: Datasets of narrow thermal hysteresis behaviour Ti-Ni-based HT-SMAs and the predicted accumulated local effects
Source: Data Brief. 2023 Oct 13;51:109654. doi: 10.1016/j.dib.2023.109654 (PMC10630592; doi:10.1016/j.dib.2023.109654)
Supplement: Supplementary file 1 [file mmc1.docx]

Prediction of narrow HT-SMA thermal hysteresis using machine learning:

A data file

Ronald Machaka

*Idya Factory Co., Hamilton, New South Wales 2303, Australia*

**Abstract**

These data are reported in a research article entitled “Prediction of narrow HT-SMA thermal hysteresis behaviour using explainable machine learning” [1].

Table of Contents

[1. Data on Effect of Ti on HTSMA dT Prediction 2](#_Toc134129189)

[2. Data on Effect of Ni on HTSMA dT Prediction 3](#_Toc134129190)

[3. Data on Effect of Pd on HTSMA dT Prediction 4](#_Toc134129191)

[4. Data on Effect of Pt on HTSMA dT Prediction 5](#_Toc134129192)

[5. Data on Effect of V on HTSMA dT Prediction 6](#_Toc134129193)

[6. Data on Effect of Hf on HTSMA dT Prediction 7](#_Toc134129194)

[7. Data on Effect of Zr on HTSMA dT Prediction 8](#_Toc134129195)

[8. Data on Effect of Cu on HTSMA dT Prediction 9](#_Toc134129196)

[9. Data on Effect of Co on HTSMA dT Prediction 10](#_Toc134129197)

[10. Reference: 11](#_Toc134129198)

# Data on Effect of Ti on HTSMA dT Prediction

**Data**

Table 1: Predicted Data:

| **Ti Content**  **(at. %)** | **Effect on dT Prediction (Centered)** |
| --- | --- |
| 27.0 | 0.49844237 |
| 34.5 | 0.65420561 |
| 43.75 | 4.00311526 |
| 46.1 | 2.99065421 |
| 47.9 | 1.82242991 |
| 49.0 | 0.03115265 |
| 49.25 | -1.44859813 |
| 49.5 | -3.39563863 |
| 50.0 | -3.62928349 |

**Data Visualization:**


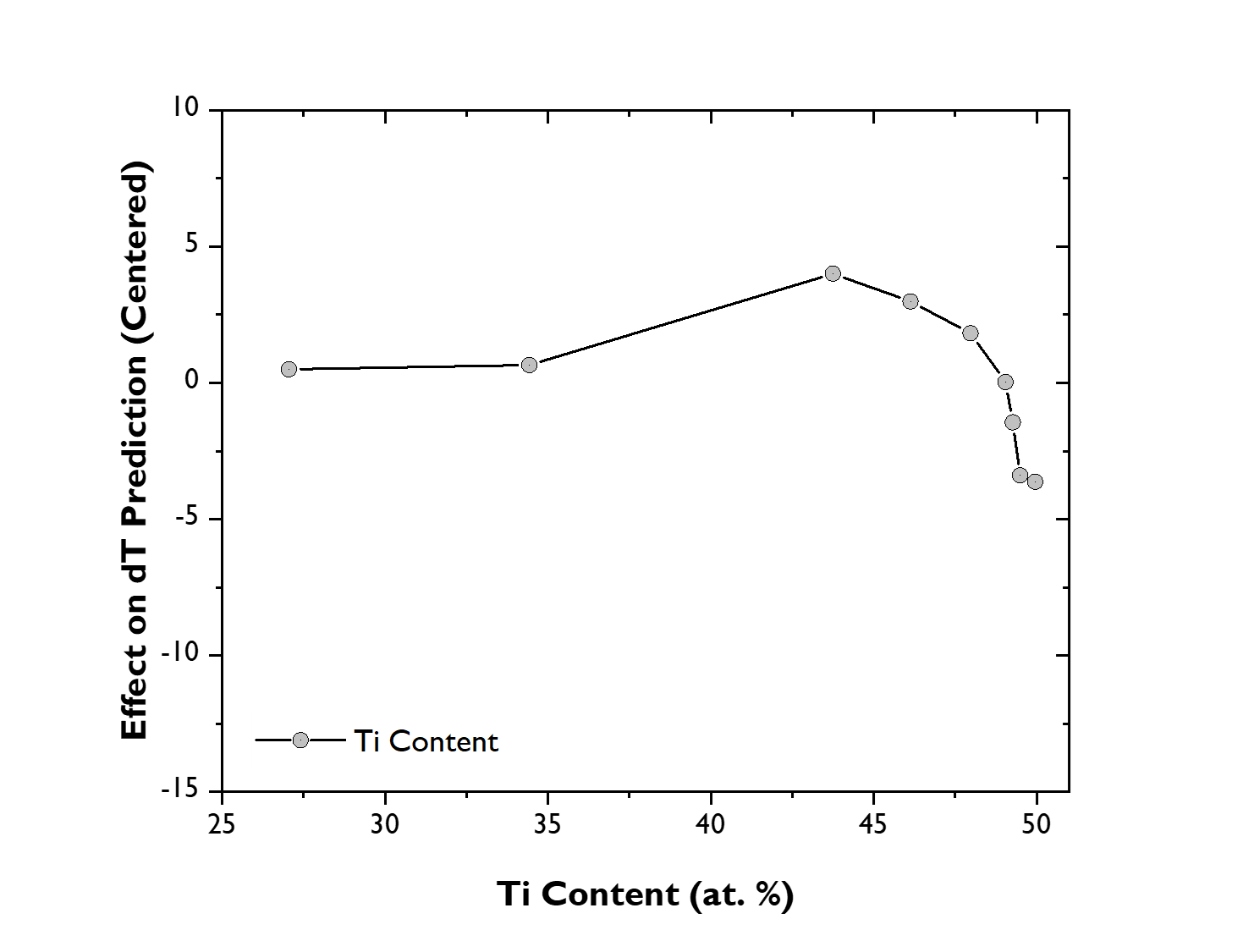


Figure 1: Predicted effect of Ti content on the HT-SMA dT Prediction [1]

# Data on Effect of Ni on HTSMA dT Prediction

**Data**

Table 2: Predicted Data:

| **Ni Content**  **(at. %)** | **Effect on dT Prediction (Centered)** |
| --- | --- |
| 0.0 | 10.4672897 |
| 18.1 | -3.78504673 |
| 27.4 | -1.76012461 |
| 35.4 | -3.86292835 |
| 38.0 | 1.12149533 |
| 41.0 | 3.30218069 |
| 42.4 | 0.03115265 |
| 50.0 | 5.732087227 |

**Data Visualization:**


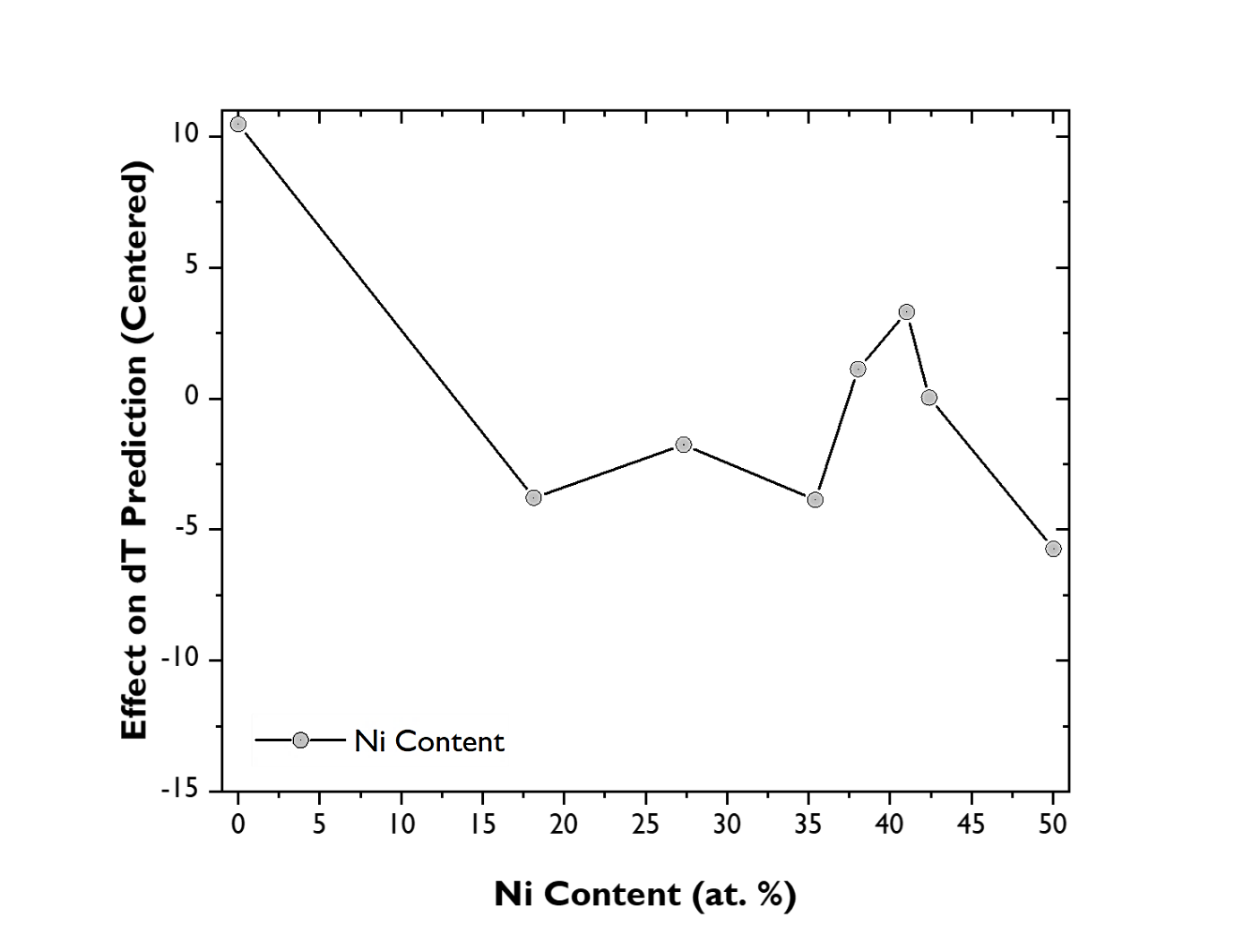


Figure 2: Predicted effect of Ni content on the HT-SMA dT Prediction [1]

# Data on Effect of Pd on HTSMA dT Prediction

**Data**

Table 3: Predicted Data:

| **Pd Content**  **(at. %)** | **Effect on dT Prediction (Centered)** |
| --- | --- |
| 0.0 | 0.18691589 |
| 5.2 | 0.42056075 |
| 8.8 | -3.08411215 |
| 11.1 | -3.08411215 |
| 17.3 | -3.70716511 |
| 25.3 | -2.77258567 |
| 42.0 | 1.82242991 |
| 50.0 | 6.96261682 |

**Data Visualization:**


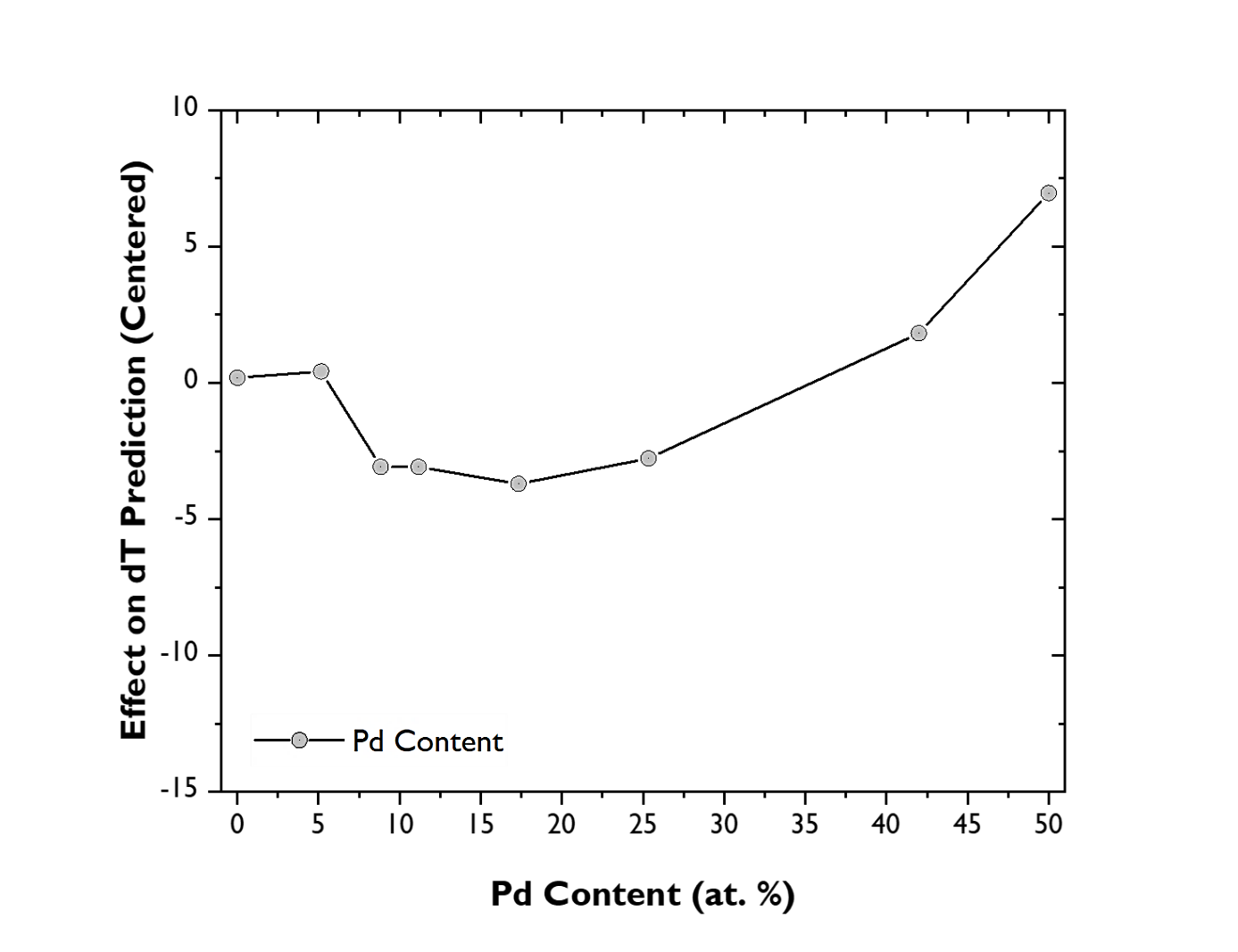


Figure 3: Predicted effect of Pd content on the HT-SMA dT Prediction [1]

# Data on Effect of Pt on HTSMA dT Prediction

**Data**

Table 4: Predicted Data:

| **Pt Content**  **(at. %)** | **Effect on dT Prediction (Centered)** |
| --- | --- |
| 0.0 | 0.06493506 |
| 5.0 | 0.12987013 |
| 18.8 | -4.15584416 |
| 42.9 | 7.07792208 |

**Data Visualization:**


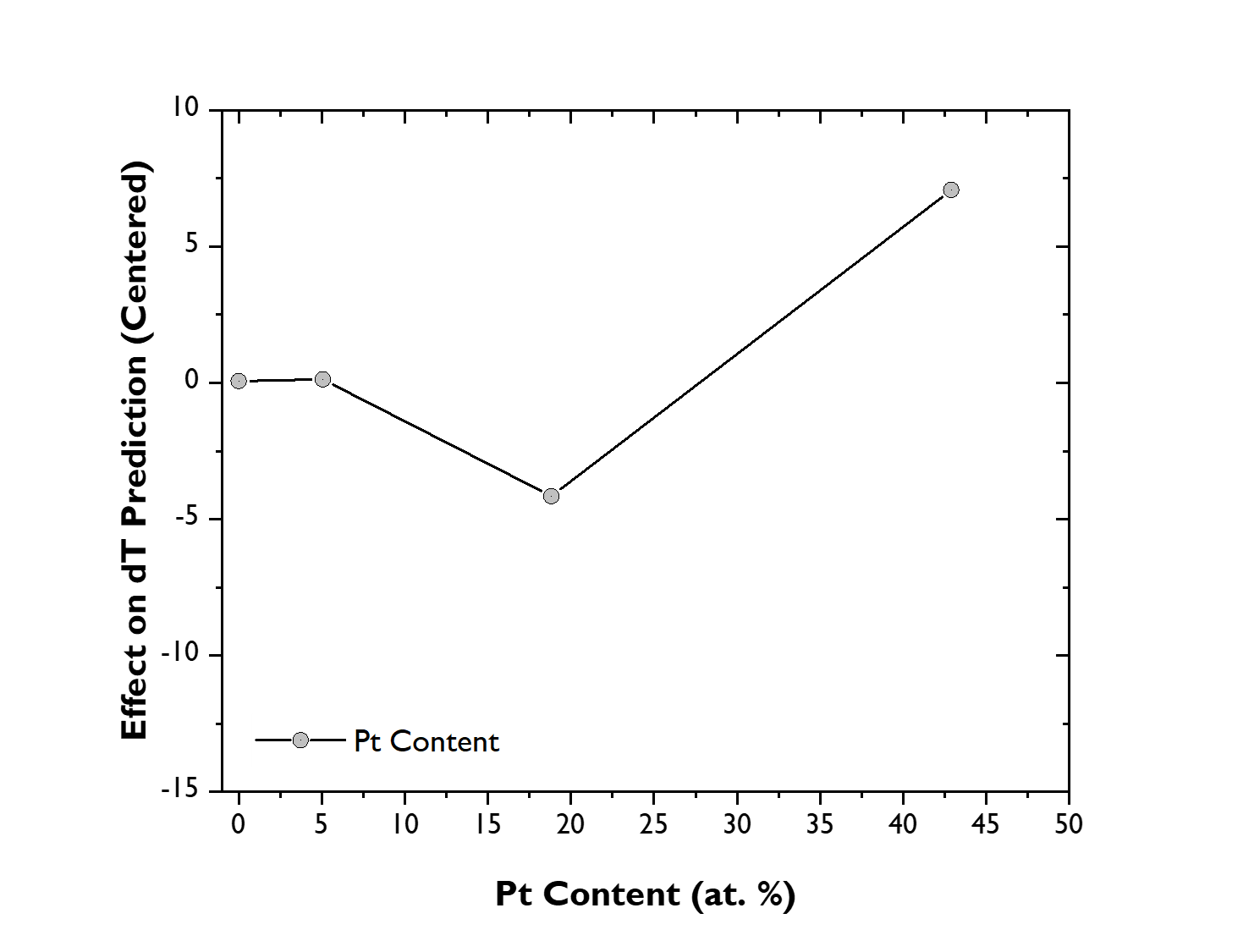


Figure 4: Predicted effect of Pt content on the HT-SMA dT Prediction [1]

# Data on Effect of V on HTSMA dT Prediction

**Data**

Table 5: Predicted Data:

| **V Content**  **(at. %)** | **Effect on dT Prediction (Centered)** |
| --- | --- |
| 0.0 | 0.34090909 |
| 0.1 | 0.19973776 |
| 0.3 | -0.36582168 |
| 1.0 | -4.26354895 |
| 5.0 | -14.7277098 |
| 6.0 | -13.0821678 |

**Data Visualization:**


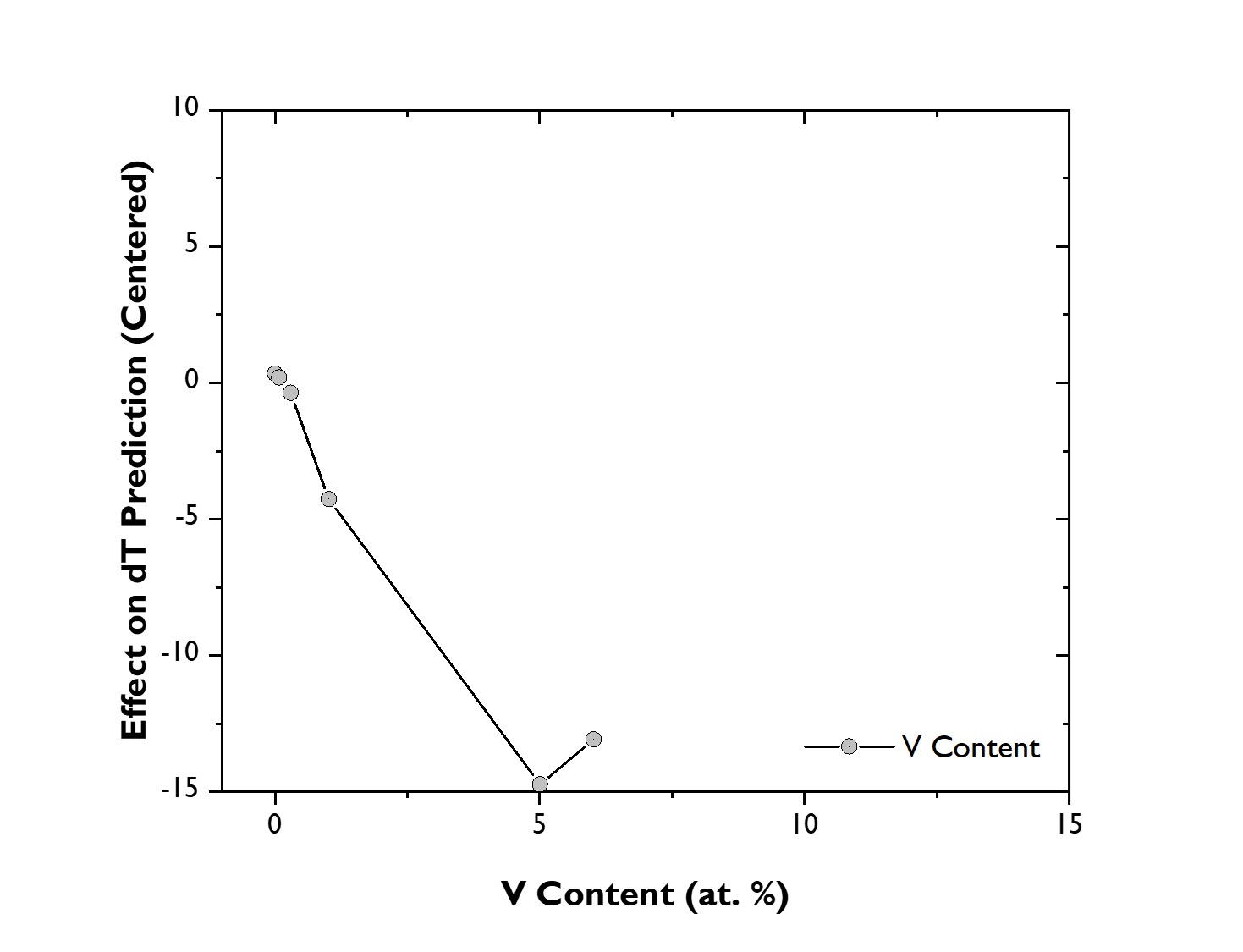


Figure 5: Predicted effect of V content on the HT-SMA dT Prediction [1]

# Data on Effect of Hf on HTSMA dT Prediction

**Data**

Table 6: Predicted Data:

| **Hf Content**  **(at. %)** | **Effect on dT Prediction (Centered)** |
| --- | --- |
| 0.0 | -1.686488 |
| 2.3 | -0.46311857 |
| 4.4 | 2.63486775 |
| 5.5 | 2.43304046 |
| 6.5 | 7.85318733 |
| 17.8 | 6.9680037 |
| 21.5 | 6.31237777 |
| 24.1 | 5.16698706 |

**Data Visualization:**


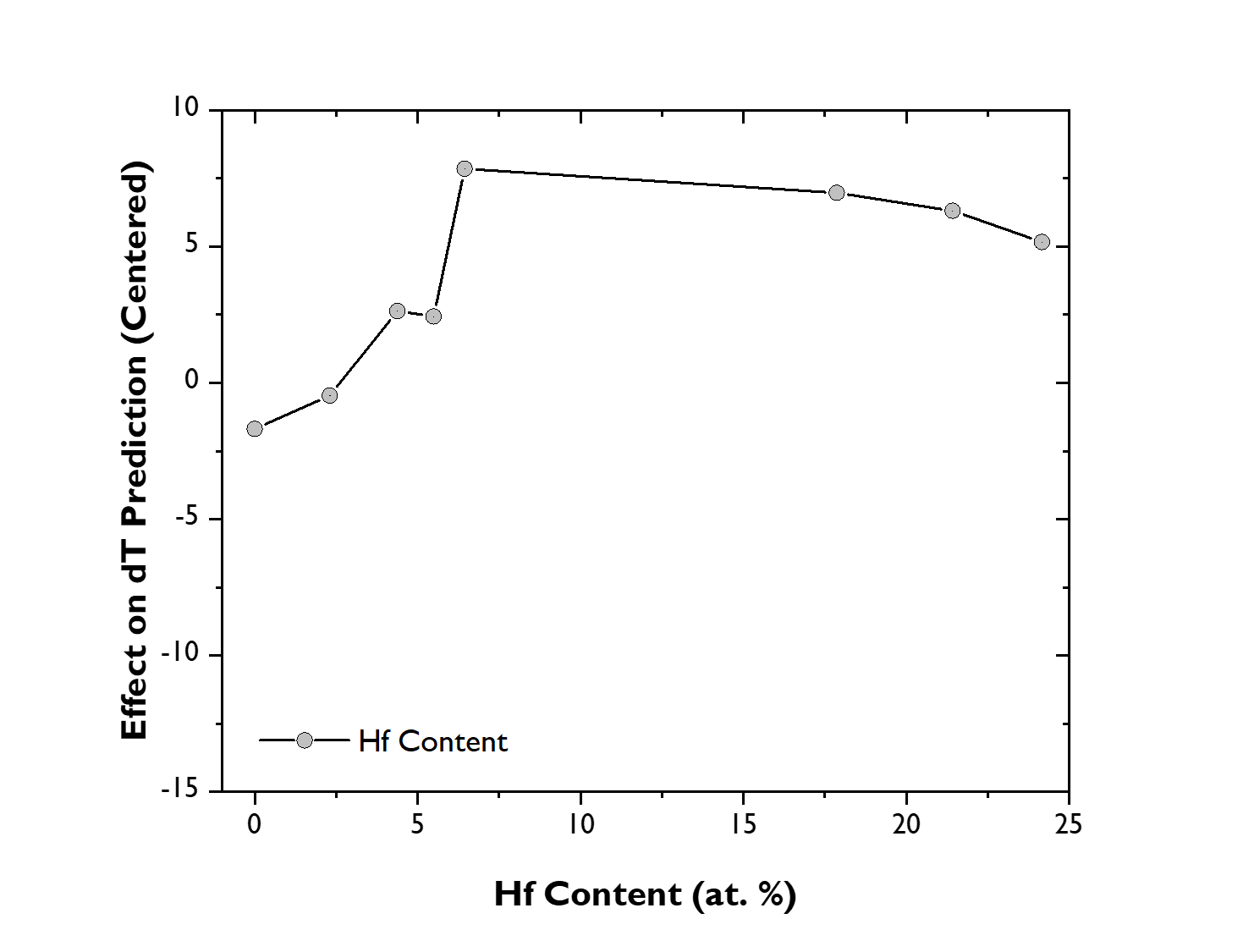


Figure 6: Predicted effect of Hf content on the HT-SMA dT Prediction [1]

# Data on Effect of Zr on HTSMA dT Prediction

**Data**

Table 7: Predicted Data:

| **Zr Content**  **(at. %)** | **Effect on dT Prediction (Centered)** |
| --- | --- |
| 0.0 | -3.42327951 |
| 1.0 | 0.69985321 |
| 3.0 | 5.11009412 |
| 5.0 | 6.253346 |
| 6.9 | 6.89944737 |
| 10.0. | 7.83352042 |
| 15.0 | 7.92224333 |
| 16.6 | 7.43092134 |
| 20.0 | 6.44870909 |

**Data Visualization:**


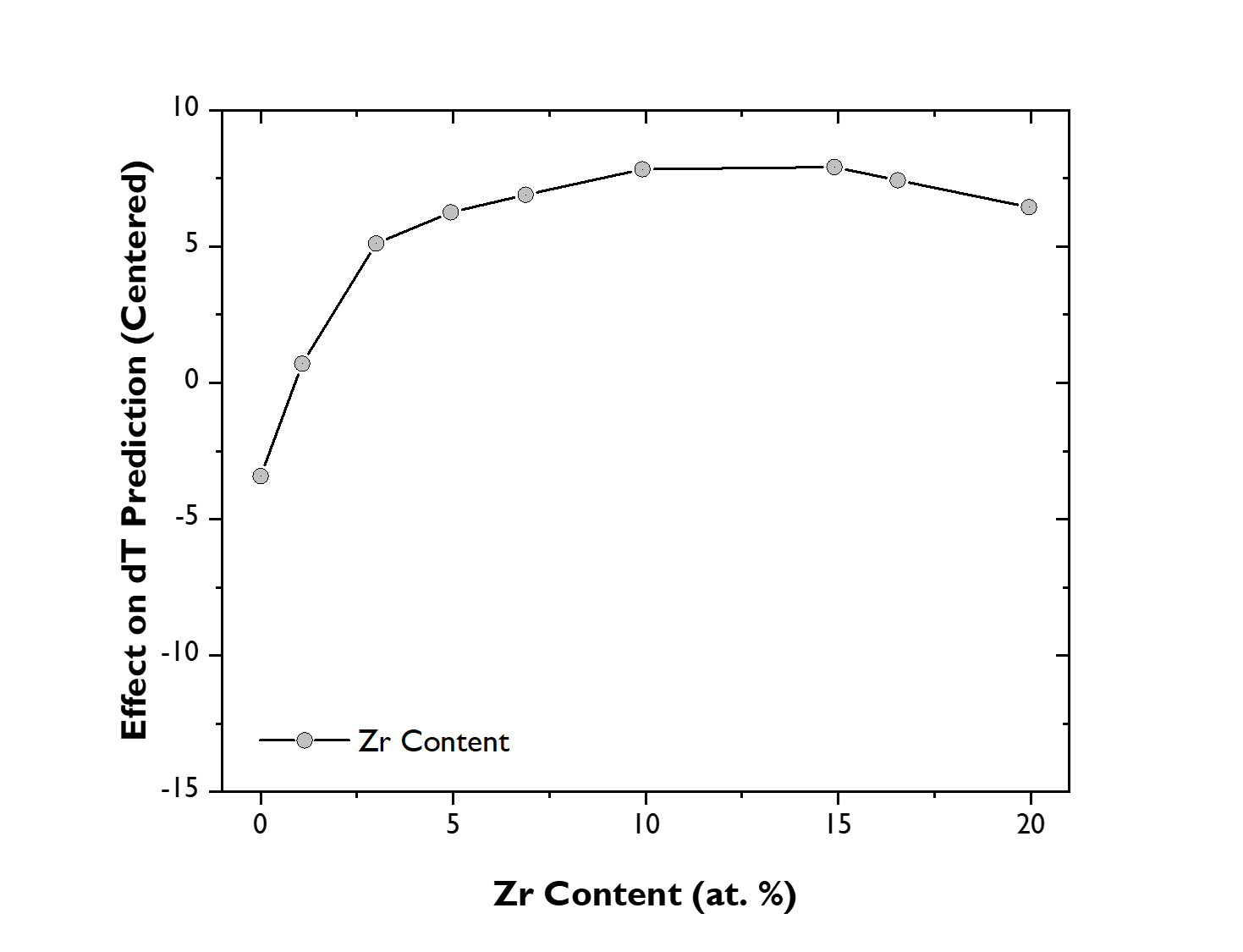


Figure 7: Predicted effect of Zr content on the HT-SMA dT Prediction [1]

# Data on Effect of Cu on HTSMA dT Prediction

**Data**

Table 8: Predicted Data:

| **Cu Content**  **(at. %)** | **Effect on dT Prediction (Centered)** |
| --- | --- |
| 0.0 | 1.19318182 |
| 2.4 | 0.90909091 |
| 2.5 | -1.78977273 |
| 3.0 | -0.58238636 |
| 5.0 | -2.21590909 |
| 7.5 | -2.21590909 |
| 9.0 | -2.42897727 |
| 11.5 | -2.21590909 |
| 14.0 | -1.78977273 |
| 15.0 | -1.22159091 |

**Data Visualization:**


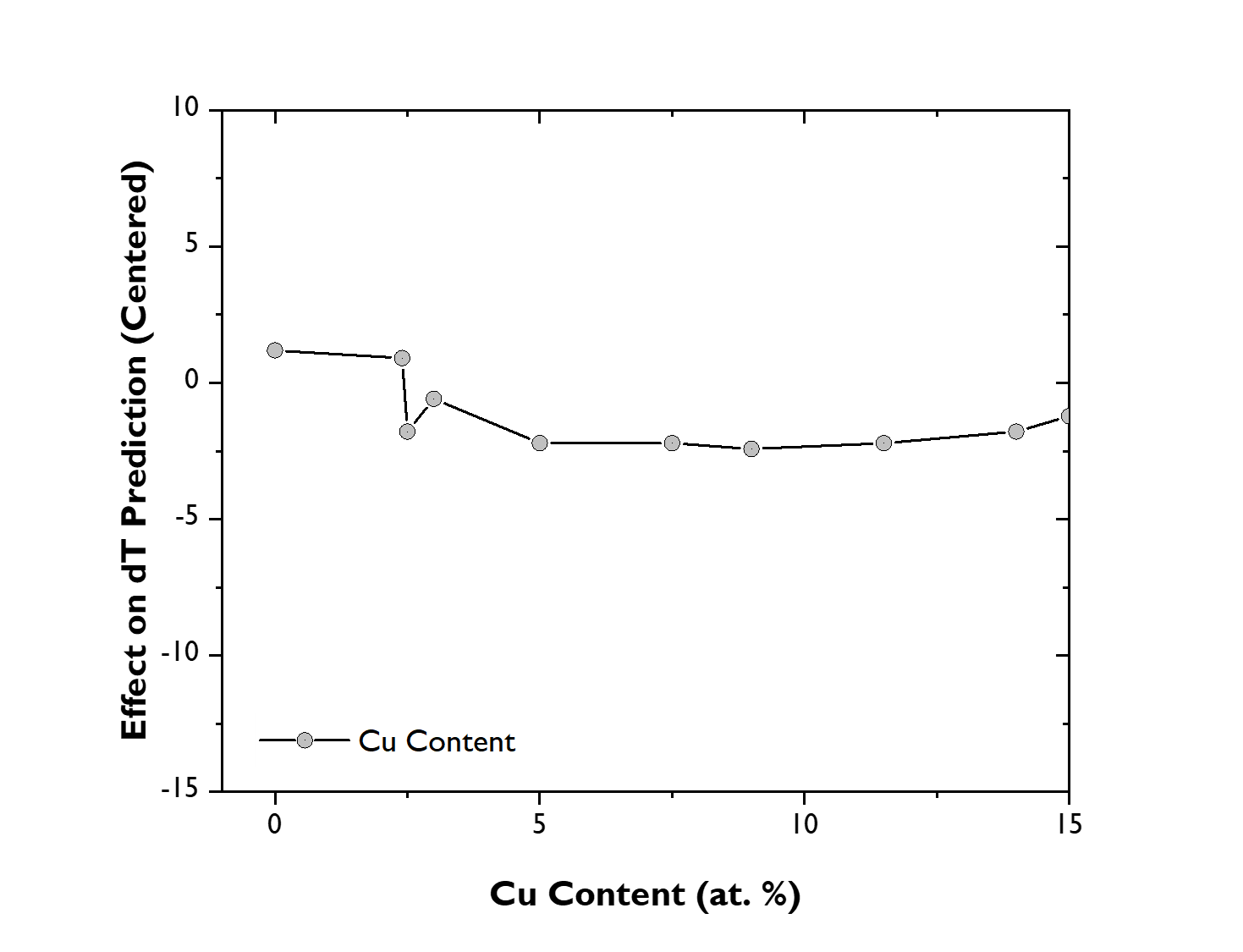


Figure 8: Predicted effect of Cu content on the HT-SMA dT Prediction [1]

# Data on Effect of Co on HTSMA dT Prediction

**Data**

Table 9: Predicted Data:

**Data Visualization:**


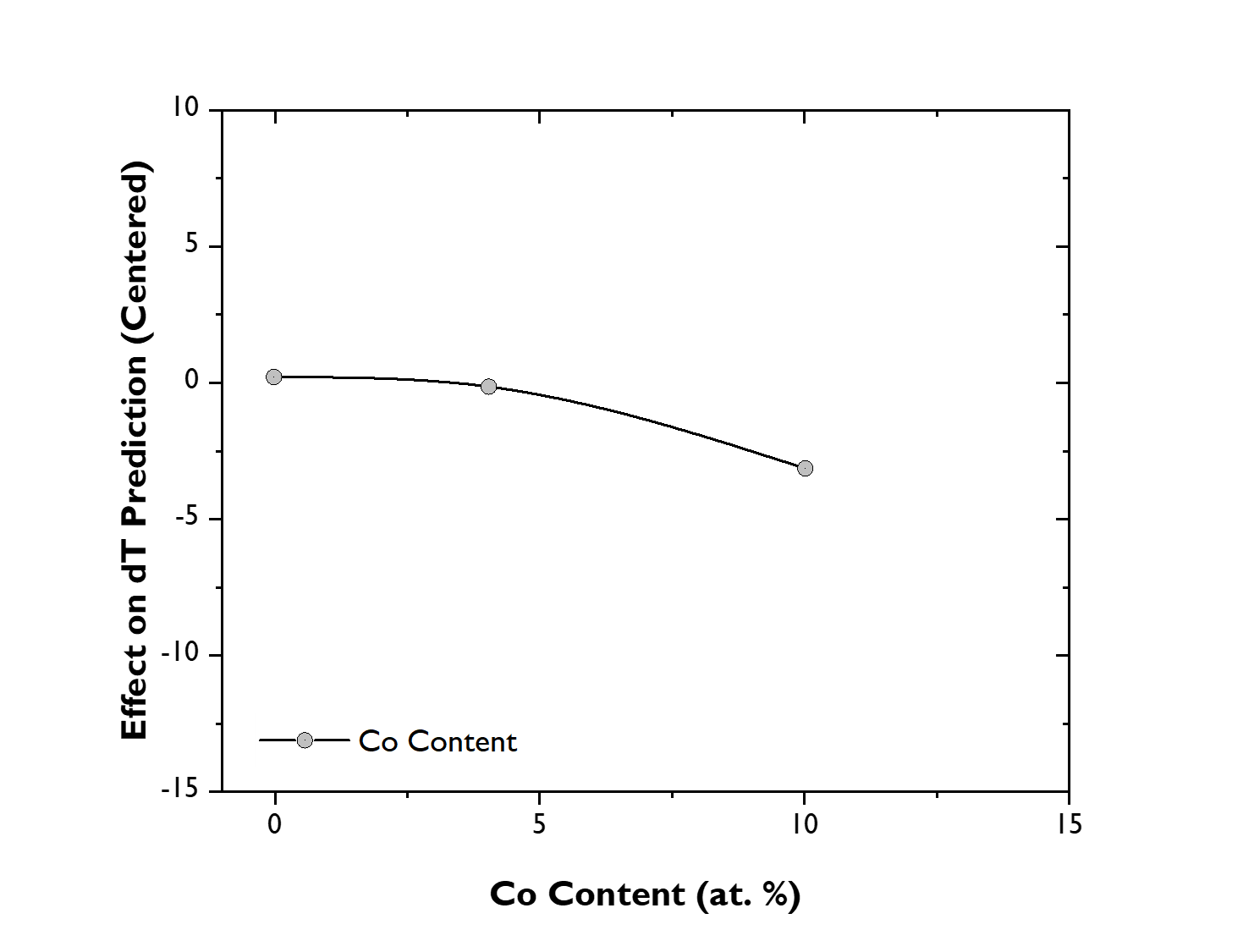


Figure 9: Predicted effect of Co content on the HT-SMA dT Prediction [1]

# Reference:

1. Ronald Machaka, Precious M. Radingoana, Prediction of narrow HT-SMA thermal hysteresis behaviour using explainable machine learning, *Mater. Today Commun.* 35 (2023) 105806. https://doi.org/10.1016/j.mtcomm.2023.105
